# Atlastin‐1 modulates seizure activity and neuronal excitability

**DOI:** 10.1111/cns.13258

**Published:** 2019-11-14

**Authors:** Xi Lu, Min Yang, Yong Yang, Xue‐Feng Wang

**Affiliations:** ^1^ Department of Neurology The First Affiliated Hospital of Chongqing Medical University Chongqing Key Laboratory of Neurology Chongqing China; ^2^ Department of Neurology The First Affiliated Hospital of Nanchang University Nanchang China

**Keywords:** epilepsy, human patients, inhibitory synaptic transmission, neuronal excitability, seizure

## Abstract

Epilepsy is a neurological disease, and the main clinical manifestation is recurrent seizures. The exact etiology of epilepsy and the pathogenesis of the disorder are not yet fully understood. Atlastin‐1, a dynamin‐like GTPase, interacts with microtubules and is responsible for vesicle formation, both of which are highly associated with the development of epilepsy. Here, we reported that the expression level of atlastin‐1 protein was reduced in the temporal neocortex of patients with temporal lobe epilepsy and in the hippocampus and adjacent cortex of a pentylenetetrazol‐kindled epileptic mouse model. Cells expressing atlastin‐1 coexpressed the inhibitory synaptic marker GAD67 in the temporal cortex and hippocampus of patients with epilepsy and an epileptic mouse model. The lentivirus‐mediated overexpression of atlastin‐1 protein in the hippocampus of mice suppressed seizure activity in behavioral experiments. Patch‐clamp recordings in the Mg^2+^‐free epilepsy cell model showed that atlastin‐1 overexpression inhibited neuronal excitability by suppressing the discharge frequency of spontaneous action potentials rather than by changing the passive and active properties of action potentials. Inhibitory synaptic transmission, but not excitatory synaptic currents, increased after atlastin‐1 overexpression. These findings suggest that atlastin‐1 likely contributes to the occurrence and development of epilepsy through inhibitory synaptic transmission.

## 1 INTRODUCTION

Epilepsy is a devastating and serious neurological disease that is characterized by recurrent unprovoked seizure activity.^1^ Almost 30% of newly diagnosed patients with epilepsy respond poorly to antiepileptic drugs and eventually develop intractable epilepsy, such as temporal lobe epilepsy.^2^, ^3^ It is well‐known that epileptic seizures are attributed to abnormal neuronal excitability due to an imbalance in excitatory and/or inhibitory synaptic transmission.^2^ However, the exact etiology of epilepsy and the pathogenesis of these disorders are not yet fully understood. Investigating the development of epilepsy will hopefully improve therapeutic strategies.

Atlastin‐1 is a new member of the dynamin/Mx/guanylate‐binding protein superfamily of large GTPases. The atlastin‐1 protein is predominantly localized in the brain and is prominently enriched in the hippocampus, mainly in pyramidal neurons in CA1 and CA3.^4^ Atlastin‐1 regulates endoplasmic reticulum (ER) and Golgi morphogenesis by controlling microtubule stability in Drosophila.^5^ In cultured rat cerebral cortical neurons, atlastin‐1 interacts with microtubules.^6^ Atlastin‐1 is also involved in dendrite and axon formation and elongation in cultured mouse cerebral cortex.^4^, ^7^, ^8^, ^9^ In addition, Drosophila atlastin‐1 is involved in vesicle budding from ER‐derived microsomes and vesicle transport in the ER‐Golgi interface.^10^ These studies indicate that atlastin‐1 may be essential for neuronal development. It is suggested that the dysregulation of microtubules and vesicle transport facilitates the occurrence and spread of epileptic discharges.^11^, ^12^, ^13^ Nevertheless, the role of atlastin‐1 in the development of epilepsy is largely unknown.

In the present study, we explored the expression of atlastin‐1 protein in patients with epilepsy and a mouse model. Furthermore, we investigated behavioral and electrophysiological changes after the lentivirus (LV)‐mediated overexpression of atlastin‐1 in the hippocampus of mice to explore the possible roles of atlastin‐1 in epilepsy.

## 2 METHOD

### 2.1 Ethics statement

All study protocols involving human subjects were approved by the Commission of Chongqing Medical University according to the guidelines of the Declaration of Helsinki. Informed consent for using human brain tissue was obtained from human subjects. All animal protocols for treatments were conducted according to the Commission of Chongqing Medical University for Ethics in Animal Experiments.

### 2.2 Human tissues

Temporal cortical tissues from 18 patients with epilepsy and 12 control individuals were obtained randomly from our human brain tissue bank (Table 1, 2, 3).^11^, ^14^, ^15^ Patients with epilepsy were diagnosed as temporal lobe epilepsy (TLE), as determined based on the 2001 International Classification of Epileptic Seizures by the International League Against Epilepsy (ILAE).^16^

**Table 1:** Clinical details of TLE patients in the study

Patients no.	Sex	Age (y)	Duration (y)	AEDs before surgery	Resected tissue	Pathological diagnosis
1	F	27	14	PHT, VAP, CZP	RTN	G
2	M	24	5	VPA, TPM,	RTN	G
3	F	15	3	CBZ, TPM, LTG	RTN	G
4	M	47	29	VPA, PHT, TPM, PB,LTG	RTN	G
5	M	28	3	PB, CBZ, VPA	LTN	NL, ND, G
6	F	38	12	CBZ, TPM, VPA	LTN	NL, ND, G
7	F	29	27	CBZ, VPA, LTG	RTN	NL, G
8	F	37	7	CBZ, TPM, VPA	LTN	NL, G
9	M	32	19	CBZ, TPM, PB,VPA	LTN	ND, G
10	M	30	10	CBZ, VPA, PHT	LTN	NL, ND, G
11	F	11	4	VPA, CBZ, TPM, LTG	RTN	G
12	M	22	7	VPA, TPM, LTG	LTN	NL, G
13	F	44	12	VPA, PHT, LTG	RTN	NL, G
14	M	33	9	VPA, PHT, LTG	RTN	G
15	M	17	3	PHT, PB, CBZ, LTG	LTN	NL, G
16	M	35	4	CBZ, VPA, PB	LTN	NL, ND
17	M	24	3	VPA, TPM, OXC	RTN	G
18	F	33	10	CBZ, PHT	RTN	G

Abbreviations: AEDs, antiepileptic drugs; C, control; CBZ, carbamazepine; CZP, clonazepam; E, epilepsy; F, female; G, gliosis; LTG, lamotrigine; LTN, left temporal neocortex; M, male; N, normal; ND, neuron degeneration; NL, neuron loss; OXC, oxcarbazepine; PB, phenobarbital; PHT, phenytoin; RTN, right temporal neocortex; TPM, topiramate; VPA, valproate.

**Table 2:** Clinical details of control patients in the study

Patients no.	Sex	Age (y)	Etiology diagnosis	Resected tissue	Pathological diagnosis
1	F	14	None	LTN	N
2	F	25	None	RTN	N
3	F	32	None	RTN	N
4	F	26	None	LTN	N
5	M	21	None	LTN	N
6	M	54	None	LTN	N
7	F	38	None	RTN	N
8	M	50	None	RTN	N
9	M	43	None	LTN	N
10	F	33	None	LTN	N
11	M	31	None	RTN	N
12	F	15	None	LTN	N

Abbreviations: F, female; LTN, left temporal neocortex; M, male; N, normal; RTN, right temporal neocortex; Y, years.

**Table 3:** Comparison of clinical data in patients of TLE and control group

Variable	TLE group	Control group	P value
Sample size	18	12	NA
Age(y)			
Range	11‐47	14‐54	NA
Mean ± SD	29.222 ± 9.247	31.833 ± 12.199	0.525
M/F ratio	10:8	5:7	0.710
Duration(y)			
Range	3‐27	NA	NA
Mean ± SD	10.056 ± 7.699	NA	NA

Abbreviations: F, female; M, male; NA, not applicable; TLE, temporal lobe epilepsy.

Control individuals underwent surgery for trauma‐induced intracranial hypertension. Temporal cortical tissues from control individuals were obtained only for treatment purposes. The control patients had no history of neurological or psychiatric disorders and had presented with no previous seizures and were not being treated with antiepileptic drugs.

### 2.3 Mouse models of pentylenetetrazol‐kindled chronic epilepsy model

Mice were kindled by pentylenetetrazol (PTZ) to develop a chronic epileptic mouse model according to previously described methods.^17^ Adult C57/BL6 male mice were administered an intraperitoneal injection of a subconvulsive dose of PTZ (35 mg/kg dissolved in 0.9% NaCl pH 7.0) every other day for 30 days and then observed for one hour after each injection. Behavioral seizure scores were evaluated according to the Racine standard scale: grade 0, arrest and normal behavior; grade 1, facial twitches (nose, lips, and eyes); grade 2, chewing and head nodding; grade 3, forelimb clonus; grade 4, rearing and falling on forelimbs and hind limb clonus; and grade 5, rearing and falling on the side or back.^18^ Given that it is technically difficult to distinguish between some normal activities and spontaneous episodes in mice, only grades 4 and 5 were considered fully kindled seizures and were included in the number of seizures. The days until full kindling were recorded as the latency period.

### 2.4 Western blot analysis

Frozen human brain tissues and the hippocampus of PTZ‐kindled mice and control mice were collected and extracted for total protein analysis using a RIPA protein extraction kit (Beyotime Biotechnology). Protein was then boiled at 95°C for 5 minutes following the calculation of protein concentrations by an enhanced bicinchoninic acid (BCA) protein assay kit (Beyotime Institute of Biotechnology). Protein was separated on 10% SDS‐PAGE gels, and the separated target proteins were immunoblotted with primary antibodies, atlastin‐1 (rabbit, Abcam), and GADPH (rabbit, Proteintech), overnight at 4°C. The blots were then incubated with HRP‐conjugated secondary antibodies. The blots were scanned and quantified using a fusion imaging system.

### 2.5 Immunofluorescence labeling

Fresh human and animal brain tissues were postfixed for 24 hours at 4°C. Floating slices (10 μm in thickness) were permeabilized with 0.5% Triton X‐100 for 30 minutes at room temperature and then incubated and blocked in goat serum. The slices were incubated with primary antibodies, atlastin‐1 (rabbit, Abcam), GAD67 (mouse, SYSY), and MAP2 (guinea pig, SYSY) overnight at 4°C followed by incubation with secondary fluorescent‐conjugated antibodies for 2 hours at room temperature. Images were captured by laser‐scanning confocal microscopy (Nikon A1 + R Microsystems) on an Olympus IX 70 inverted microscope (Olympus). Areas of fluorescence intensity were analyzed using Image Pro Plus 6.0.

### 2.6 Intrahippocampal injection of LV

Healthy adult C57/BL6 male mice weighing 20‐30 g were provided by the Experimental Animal Center of Chongqing Medical University. Intrahippocampal injections of lentiviral vector were administered by a stereotaxic apparatus (Stoelting Co. Ltd). Briefly, the mice were anesthetized with pentobarbital (80 mg/kg, intraperitoneal) and then placed in a stereotaxic apparatus. The reference points were the dura mater for the dorsal‐ventral axis, bregma for the anterior‐posterior axis, and the midline for the medial‐lateral axis. Lentiviral vector particles were injected into the hippocampus by a glass pipette (0.2 μL/min) attached to a glass microsyringe.^14^ The injected mice were allowed to fully recover for 2 weeks to prevent infection, and recovery was followed by PTZ injection for behavioral observation.

### 2.7 Patch‐clamp recordings

Brain slices of adult C57/BL6 male mice were obtained as previously reported.^19^, ^20^ Briefly, 2 weeks after the intrahippocampal injection of LV, mice were anesthetized with pentobarbital (80 mg/kg, intraperitoneal). Brain slices (300 μm) were prepared with a Leica (Germany) VP1200S Vibratome. Slices were then incubated in artificial cerebral spinal fluid (ACSF) (119 mmol/L NaCl, 26 mmol/L NaHCO_3_, 2 mmol/L CaCl_2_, 2.5 mmol/L KCl, 1.25 mmol/L NaH_2_PO_4_, 1 mmol/L MgCl_2,_ and 25 mmol/L glucose, pH 7.4, 300‐310 mOsm) at room temperature for at least 1 hour before recording. In the meantime, ACSF was continuously bubbled with 5% CO_2_ and 95% O_2_. Glass microelectrodes (Sutter) were prepared by a pipette puller to record a final resistance of 3‐5 MΩ when the pipette was filled with an internal solution. A multiclamp 700B amplifier (Axon) was used in whole‐cell recording.^21^ The obtained signals were sampled at 10 kHz and filtered at 2 kHz. A stable baseline was obtained for at least 5 minutes prior to recording. The access resistance was checked intermittently in current‐clamp mode, and the data were abandoned if the access resistance (15‐20 MΩ) had changed by 20% at the end of recording. Mini Analysis 6.0.1 (Synaptosoft) and Clamp Fit 10.3 software (Axon) were used to analyze the data.

For action potentials (APs) recordings, glass microelectrodes were tip‐filled with 0.5 μl normal solution: 17.5 mmol/L KCl, 122.5 mmol/L K‐gluconate, 0.5 mmol/L EGTA, 10 mmol/L HEPES, and 4 mmol/L ATP, 7.2 pH. The internal solution used to record excitatory postsynaptic currents (EPSCs) contained the following: 17.5 mmol/L CsCl, 0.5 mmol/L EGTA, 132.5 mmol/L Cs‐gluconate, 10 mmol/L HEPES, 4 mmol/L ATP, and 5 mmol/L QX‐314. The internal solution used to record inhibitory postsynaptic currents (IPSCs) contained the following: 100 mmol/L CsCl, 1 mmol/L EGTA, 30 mmol/L N‐methyl‐d‐glucamine, 1 mmol/L MgCl_2_, 5 mmol/L MgATP, 0.5 mmol/L Na_2_GTP, 12 mmol/L phosphocreatine, and 10 mmol/L HEPES. Tetrodotoxin (TTX, 1 μmol/L) and bicuculline (10 μmol/L) were added to Mg‐free ACSF to record mEPSCs. TTX (1 μmol/L), APV (40 μmol/L), and DNQX (20 μmol/L) were added to Mg‐free‐ACSF to record mIPSCs.^14^

Passive (resting membrane potential and input resistance) and active (AP duration, overshoot, and afterhyperpolarization) electrophysiological properties were monitored as previously described.^22^, ^23^ Briefly, the input resistance was determined by current injection in response to a hyperpolarizing voltage step from −70 mV. Spontaneous activity was assessed by observing the neuron under current clamp for 1 minute, after which active electrophysiological properties were determined. A 500 ms depolarizing rectangular current injection was used to assess AP duration, AP overshoot, and afterhyperpolarization.

Paired‐pulse ratios (PPRs) recordings were voltage‐clamped at −70 mV using a paired‐pulse protocol of two stimuli at an interpulse interval of 50 ms PPRs were calculated by the ratio of the second peak amplitude to the first peak amplitude.

### 2.8 Statistical analysis

**Figure 1:**
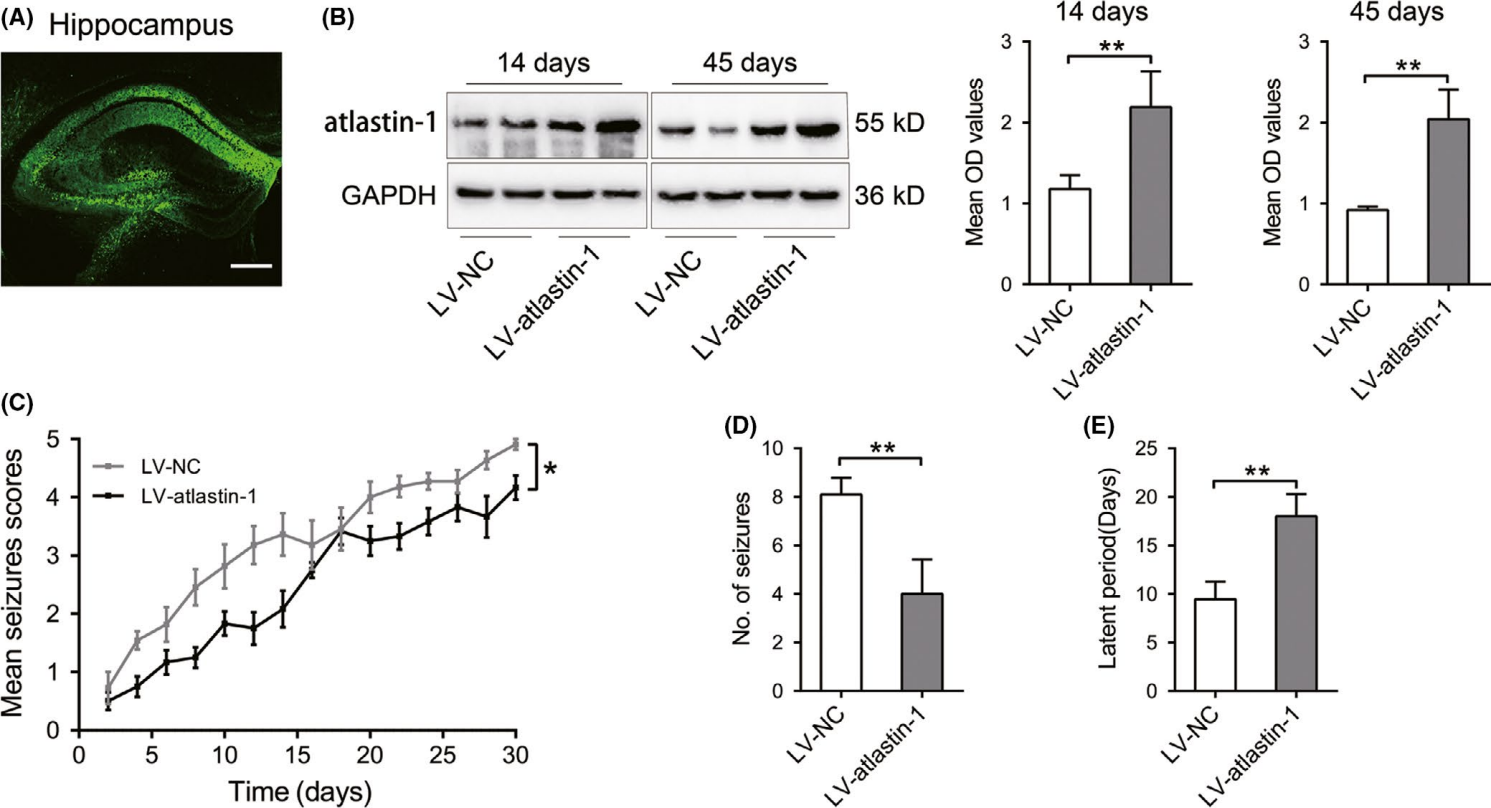
Effect of LV‐atlastin‐1 on seizure activity in PTZ‐kindled mice. A, lentiviral‐encoding GFP was detected in the mouse hippocampus after an injection of LV. Bar, 250 μm. B, hippocampal atlastin‐1 protein expression levels were detected in atlastin‐1 overexpressing mice 14 and 45 d after LV injection. n = 5 mice in each group. C‐E, quantitative analysis of seizure grade (C), total number of seizures with a grade 4 or 5 (D), and latency of the first full kindling seizure with a grade of 4 or 5 (E) in PTZ‐kindled mice after atlastin‐1 overexpression. n = 7‐10 mice in each group. **P < .01

Student's *t*‐test was used for independent‐samples. Repeated measures ANOVA followed by post hoc t‐tests were used to compare the differences in the daily seizure scores of PTZ‐kindled mice (Figure 1C). The chi‐square test was used to compare gender differences between TLE patients and controls. All data are presented as the mean ± SD *P* < .05 and *P* < .01 were considered statistically significance. n indicates the number of mice/slices or independent experiments. All samples included in each experiment were analyzed in triplicate. All graphs were prepared using GraphPad Prism 4 software.

## 3 RESULTS

### 3.1 Reduced atlastin‐1 protein in TLE patients

**Figure 2:**
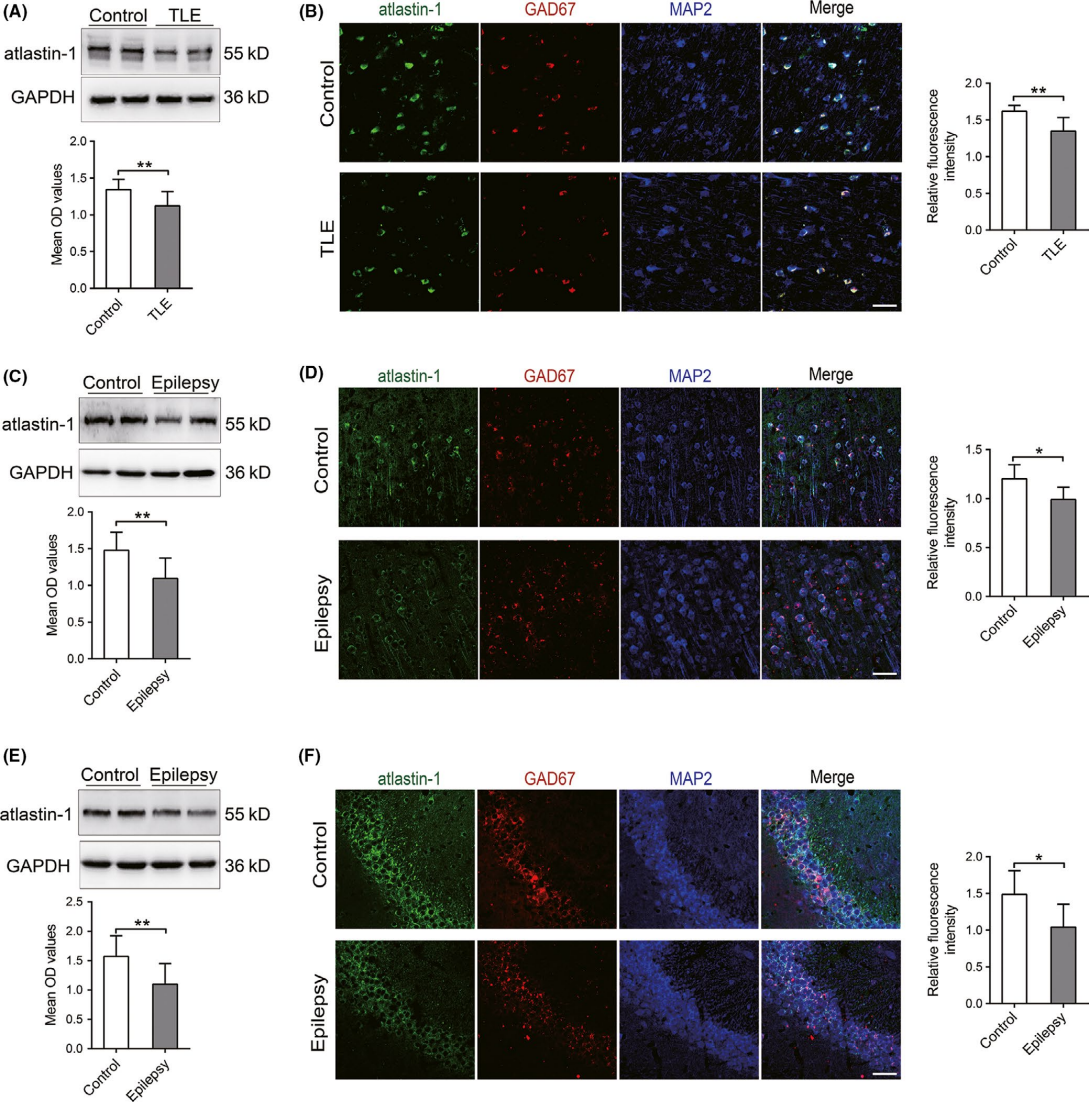
Expression of atlastin‐1 in TLE patients and epileptic mice. A,B, atlastin‐1 expression in the temporal neocortex of patients with TLE (n = 18) and control patients (n = 12) by immunoblot (A) and immunofluorescence staining (B). Bar, 50 μm. C,D, atlastin‐1 expression in the temporal cortex in PTZ‐kindled mice by immunoblot (C) and immunofluorescence staining (D). E,F, atlastin‐1 expression in the hippocampal CA3 region in PTZ‐kindled mice by immunoblot (E) and immunofluorescence staining (F). Atlastin‐1 was colocalized with the inhibitory postsynaptic marker GAD67, and MAP2 is the neuronal marker (D,F). n = 6 mice in each group. Bar, 50 μm. OD, optical density. *P < .05, **P < .01

The expression patterns of atlastin‐1 protein in the temporal lobe neocortex of patients with TLE and controls were detected by western blot and immunofluorescence labeling. The expression level of atlastin‐1 protein in TLE patients was significantly lower than that in controls according to western blot (Figure 2A). By immunofluorescence, atlastin‐1 protein was mainly found in the neuronal cytoplasm. Cells in the temporal cortices of patients with epilepsy expressing atlastin‐1 coexpressed the inhibitory synaptic marker GAD67 (Figure 2B). Compared with controls, atlastin‐1 protein in TLE patients was found to have a low fluorescence intensity in quantitative immunofluorescence analysis (Figure 2B).

### 3.2 Decreased atlastin‐1 protein in an epileptic mouse model

Atlastin‐1 protein in the PTZ‐kindled epileptic mouse model was further analyzed by Western blot and quantitative immunofluorescence. The expression results in the mouse model were similar to the expression results in humans. Atlastin‐1 protein levels were significantly lower in both the neocortex (Figure 2C) and hippocampus (Figure 2E) of PTZ‐kindled mice than in the corresponding regions in controls. Additionally, in quantitative immunofluorescence analysis, weak green staining was detected for atlastin‐1 protein in the cortex (Figure 2D) and hippocampus (Figure 2F) of PTZ‐kindled mice, whereas strong staining for atlastin‐1 was detected in the corresponding regions of controls. In addition, atlastin‐1 protein was mainly colocalized with the inhibitory synaptic marker GAD67 in the cortex and hippocampus of epileptic mice (Figure 2D,F).

### 3.3 Identify atlastin‐1 overexpression in the mouse hippocampus

To explore the possible role of atlastin‐1 in epilepsy, we first validated the efficiency and duration of the effects of recombinant lentivirus (LV)‐mediated atlastin‐1 overexpression in mice. The transfection efficiency of LV was detected at 14 and 45 days after LV injection into the dorsal hippocampal area of mice. In Figure 1A, green fluorescent protein (GFP) autofluorescence staining was shown in the injected hippocampus. By Western blot, the expression levels of atlastin‐1 were significantly increased in the atlastin‐1 overexpression group compared with the corresponding negative control(NC) group on days 14 and 45 after LV injection (Figure 1B). These results suggested that the lentiviral vector was successfully infected into hippocampal neurons and efficiently increased the expression level of atlastin‐1 protein.

### 3.4 Hippocampal overexpression of atlastin‐1 protein suppressed seizure activity in a PTZ‐induced epileptic mouse model

To further explore the potential relationship between atlastin‐1 protein and the pathology of epilepsy, behavioral effects related to atlastin‐1 protein were detected in the epileptic mouse model. In PTZ‐induced epileptic mice, behavioral observation showed that the daily seizure scores across 30 days were lower in atlastin‐1 overexpressing mice than in controls (Figure 1C). Compared with the controls, atlastin‐1 overexpressing mice exhibited a lower total number of seizures (Figure 1D) and a longer induced latency (Figure 1E). These behavioral results suggested that atlastin‐1 reduced epileptic seizure activity through the regulation of the severity, frequency, and latency of seizures.

### 3.5 Atlastin‐1 inhibited neural excitability in the Mg^2+^‐free epilepsy cell model

**Figure 3:**
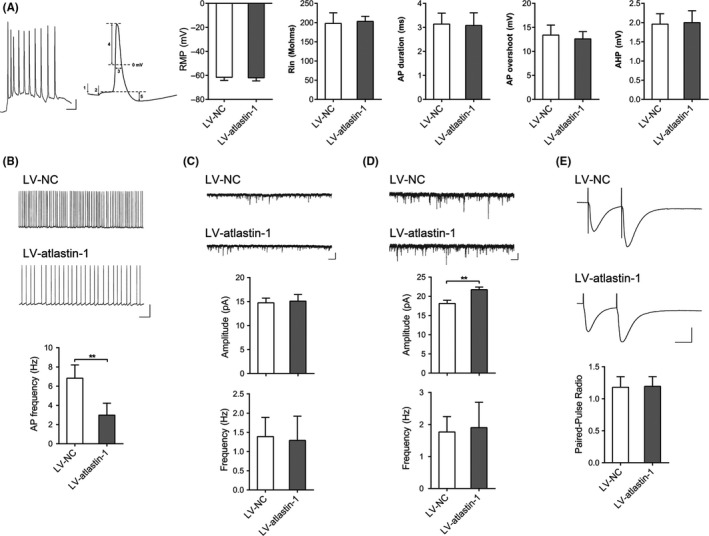
Effects of atlastin‐1 on electrophysiology in mouse hippocampal brain slices. A, representative traces of APs evoked by injecting depolarizing currents. Bar, 0.1 s, 10 mV. The passive and active electrophysiological properties of APs are shown. 1, RMP (resting membrane potential), 2, Rin (input resistance), 3, AP duration, 4, AP overshoot, and 5, AHP (afterhyperpolarization). B, the frequency of spontaneous APs was compared between the LV‐atlastin‐1 group and the control group. Bar, 1 s, 25 mV. C, D, quantitative analysis of mEPSC (C) and mIPSC (D) amplitude and frequency in CA3 pyramidal neurons in the hippocampus for the LV‐atlastin‐1 group and the control group. Bar, 1 s, 20 pA. E, overexpressing atlastin‐1 did not alter paired‐pulse ratio (PPR) values. Bar, 25 ms, 80 pA. n = 5‐7 neurons from 3 mice in each group. **P < .01

To investigate the role of atlastin‐1 in neural electrophysiology, whole‐cell patch clamp was performed in pyramidal neurons of the hippocampal CA3 area in Mg^2+^‐free brain slices. We first studied the effect of atlastin‐1 on the passive and active properties of action potentials (APs).^22^, ^23^ There were no significant differences in resting membrane potential, input resistance, AP duration, AP overshoot, or afterhyperpolarization between the two groups (Figure 3A). However, the discharge frequency of spontaneous APs in the atlastin‐1 overexpressing group was lower than that in the control group (Figure 3B).

To further investigate whether atlastin‐1–mediated neuronal hypoexcitability is an imbalance between the excitatory and inhibitory synaptic currents, miniature excitatory postsynaptic currents (mEPSCs) and miniature inhibitory postsynaptic currents (mIPSCs) were recorded.^24^, ^25^ In pyramidal neurons in the CA3 region of the hippocampus, the amplitude nor the frequency of mEPSCs was significantly changed between the atlastin‐1 overexpressing group and the control group (Figure C). However, the mIPSC amplitude, but not the frequency, was increased in atlastin‐1 overexpressing mice compared with controls (Figure 3D). We then analyzed PPRs to further explore the impact of atlastin‐1 on presynaptic vesicle release.^26^ There was no significant difference in the PPRs between atlastin‐1 overexpressing mice and controls (Figure 3E).

## 4 DISCUSSION

Our study first identified that the expression level of atlastin‐1 protein was decreased in brain tissues from patients with epilepsy and epileptic mice. The overexpression of atlastin‐1 protein in the hippocampus significantly inhibited seizure activity by prolonging the latency period of seizures and suppressing seizure severity. In addition, atlastin‐1 overexpression significantly reduced spontaneous action potentials and increased inhibitory postsynaptic transmission in neurons. These results revealed a previously unknown function of atlastin‐1 protein in epilepsy, suggesting that it may be involved in the development of epilepsy.

Epilepsy is a chronic brain disease, and the mechanisms are mainly focused in the brain, which then triggers a secondary cascade of various pathology events.^1^, ^2^ Thus, brain tissue from humans is the most valuable and reliable material with which to explore clinical‐pathological alterations in epilepsy. In the present study, we first collected the temporal lobe cortex of patients with TLE to verify that atlastin‐1 protein was significantly reduced in TLE patients. Thus, atlastin‐1 protein may be closely associated with epilepsy.

Due to ethical requirements, we could not acquire normal hippocampal tissue from humans; thus, relatively normal brain tissue from patients with increased intracranial pressure due to head trauma was obtained as the control. Given that we could not equally compare the expression level of atlastin‐1 between the normal hippocampus and the pathological hippocampus of TLE, we detected the atlastin‐1 expression level in an epileptic mouse model to provide direct evidence that the atlastin‐1 levels in the hippocampus decreased during seizure activity. Notably, atlastin‐1 was expressed at significantly lower levels in the hippocampus and adjacent temporal cortex of the epileptic mice than in controls. These results further indicated that the abnormal expression of atlastin‐1 protein in the brain may be involved in epileptogenesis.

The expression results suggested that decreased atlastin‐1 protein levels might play a protective role against seizure activity. For a more in‐depth analysis, the potential causal relationship between atlastin‐1 proteins and epilepsy was determined. We used an in vivo intervention utilizing lentivirus to overexpress atlastin‐1 protein in the classic PTZ‐kindled epileptic mouse model to detect the behavioral effects of atlastin‐1 levels on seizure activity The PTZ‐kindled model employs long‐term repeated hippocampal stimulation by recurrent injections of a subthreshold dose of PTZ, which results in an imbalance of synaptic excitability and inhibitory input and increasingly raises susceptibility to seizures, ultimately leading to recurrent unprovoked seizures.^27^ This model has been well‐known to effectively screen antiepileptic drugs for epilepsy treatment. In our study, the severity of seizures was suppressed and the latency period to full kindling seizures was lengthened after the overexpression of atlastin‐1 in the mouse hippocampus. It was suggested that abnormal atlastin‐1 expression might be an etiological factor underlying epileptic seizures rather than the pathogenic result of them. Thus, an increase in atlastin‐1 protein levels in the hippocampus may protect against seizure occurrence in epilepsy.

Epileptic seizures occur as a result of the abnormally intense and highly synchronized firing of brain cells.^2^ The hippocampus is a crucial pathological locus in the development of epilepsy. Some reports indicated that the CA3 area acts as the pacemaker for the initiation of synchronous activities in neuronal populations, which spreads to recruit other regions.^28^, ^29^, ^30^, ^31^ Therefore, we recorded electrophysiological data to explore the effects of atlastin‐1 on pyramidal neurons in the CA3 area of the hippocampus to study the role of atlastin‐1 in epileptogenesis. Because the hyperexcitability of neurons in the brain is the basis of the electrophysiological changes that occur in epileptic seizure, we first investigated the pyramidal neural excitability of Mg^2+^‐free in the CA3 region of hippocampal slices after atlastin‐1 overexpression. Atlastin‐1 decreased the discharge frequency of spontaneous APs, but did not alter the passive and active properties of APs. These results indicated that specific ion channels may not be involved in the atlastin‐1–mediated changes in neuronal excitability.^22^, ^23^

mIPSCs are supposed to indicate gamma‐aminobutyric acid (GABA)‐mediated inhibitory synaptic transmission; in contrast, mEPSCs reflect glutamate‐mediated excitatory synaptic transmission.^32^ We further investigated the role of atlastin‐1 on excitability and inhibitory balance in pyramidal neurons. Without influencing mEPSCs, atlastin‐1 highly reduced neural excitability due to increased mIPSC currents. In addition, atlastin‐1 only reduced mIPSC amplitudes, but not frequencies, and it had no effect on the PPR. The frequency of postsynaptic currents reflects the number of presynaptic neurotransmitter vesicles released in a unit of time, and the amplitude of postsynaptic currents indicates the effect on the postsynaptic receptor.^33^ The PPR is also a reliable index of the contribution of presynaptic neurotransmitter vesicles release to postsynaptic currents.^26^ There was no significant difference in mIPSC frequency and PPR after atlastin‐1 overexpression, indicating that presynaptic vesicle release might not be responsible for the atlastin‐1–mediated changes in mIPSCs. Atlastin‐1–mediated changes in synaptic currents might be controlled by postsynaptic GABA receptors rather than presynaptic GABA neurotransmitter release. In addition, atlastin‐1 colocalized with the inhibitory synaptic marker GAD67 in the temporal cortex of TLE patients and PTZ‐kindled mice, further indicating its role in inhibitory synaptic transmission.

The atlastin‐1 protein is encoded by the SPG3A gene which is often found to be mutated in hereditary spastic paraplegia (HSP). HSP is a group of inherited neurodegenerative disorder characterized by progressive spasticity and weakness in the lower limbs. Complex forms of HSP exhibit additional symptoms such as seizures.^34^ Atlastin‐1 is enriched in the adult brain, but is also present at much lower levels in some other human tissues such as smooth muscle, adrenal gland, testis, lung, and kidney. In the brain, atlastin‐1 is widely expressed in the pyramidal neurons in lamina V of the cerebral cortex and in the CA1 and CA3 areas of the hippocampus,^4^ which are the most important brain regions associated with the pathogenesis of experimental epilepsy.^35^ As a dynamin‐like GTPase, atlastin regulates the stability of microtubules in Drosophila.^5^ Recent studies have identified that atlastin‐1 is implicated in vesicle budding from ER‐derived microsomes and in the vesicle transport step of the ER‐Golgi interface.^10^ Notably, as shown in previous studies, microtubule and vesicle formation are essential for the transportation of vesicles containing GABA receptors from ER‐Golgi to the plasma membrane in epilepsy.^36^, ^37^ Thus, we speculate that atlastin‐1 might be implicated in GABA receptor‐related vesicle transport in epilepsy, thereby increasing GABA receptor‐mediated inhibitory synaptic transmission and causing neural hypoexcitation.

In conclusion, we reported for the first time that atlastin‐1 plays an important role in epileptogenesis by modulating seizure activity and neuronal excitability. However, the mechanism underlying the atlastin‐1 change in inhibitory synaptic input should be further investigated.

## CONFLICTS OF INTEREST

The authors declare no competing interests.
